# Great Debate: Computed tomography coronary angiography should be the initial diagnostic test in suspected angina

**DOI:** 10.1093/eurheartj/ehac597

**Published:** 2023-03-14

**Authors:** Colin Berry, Christopher M Kramer, Vijay Kunadian, Toral R Patel, Todd Villines, Raymond Y Kwong, Daniell Edward Raharjo

**Affiliations:** British Heart Foundation Glasgow Cardiovascular Research Centre, 126 University Place, University of Glasgow, Glasgow, G128TA, UK; Golden Jubilee National Hospital, Agamemnon Street, Clydebank, G81 4DY, UK; Cardiovascular Division, Department of Medicine, University of Virginia Health System, 1215 Lee St., Box 800158, Charlottesville, VA 22908, USA; Department of Radiology and Medical Imaging, University of Virginia Health System, 1215 Lee St., Box 800170, Charlottesville, VA 22908, USA; Translational and Clinical Research Institute, Faculty of Medical Sciences, Newcastle University, 4th Floor William Leech Building, Newcastle upon Tyne NE2 4HH, UK; Cardiothoracic Centre, Freeman Hospital, Newcastle upon Tyne Hospitals NHS Foundation Trust, Newcastle upon Tyne, UK; Cardiovascular Division, Department of Medicine, University of Virginia Health System, 1215 Lee St., Box 800158, Charlottesville, VA 22908, USA; Cardiovascular Division, Department of Medicine, University of Virginia Health System, 1215 Lee St., Box 800158, Charlottesville, VA 22908, USA; Cardiovascular Division, Department of Medicine, Brigham and Women’s Hospital and Harvard Medical School, Boston, MA, USA; Translational and Clinical Research Institute, Faculty of Medical Sciences, Newcastle University, 4th Floor William Leech Building, Newcastle upon Tyne NE2 4HH, UK; Cardiothoracic Centre, Freeman Hospital, Newcastle upon Tyne Hospitals NHS Foundation Trust, Newcastle upon Tyne, UK

## Abstract

**Graphical Abstract:**
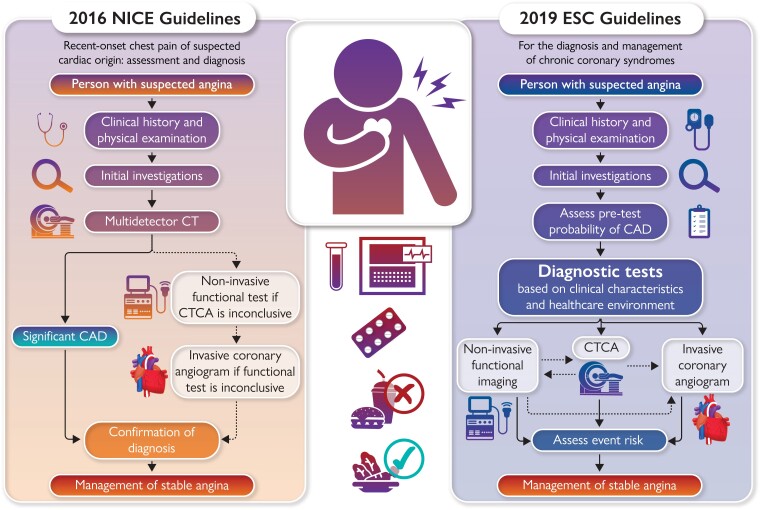

## Introduction

https://orcid.org/0000-0002-4547-8636BerryColin
British Heart Foundation Glasgow Cardiovascular Research Centre, 126 University Place, University of Glasgow, Glasgow, G128TA, UK
Golden Jubilee National Hospital, Agamemnon Street, Clydebank, G81 4DY, UK

Corresponding author. Fax: 4414143303325, Email: colin.berry@glasgow.ac.uk

Today, chest pain is one of the most common reasons for attending primary and secondary care, and the attending clinician has multiple factors to consider.^1^ A key question is whether the symptoms are of cardiac origin. If yes, the symptoms may be classified as angina (typical or atypical) or non-anginal, e.g. pericarditis. Central chest pain that occurs with effort or physiological stressors and resolves with rest represents ‘typical angina’. The classical cause is obstructive coronary artery disease (CAD) but microvascular disease may equally cause typical angina. Symptoms such as effort-related breathlessness or spontaneous chest discomfort (not associated with effort) may arise secondary to myocardial ischaemia and be classified as ‘atypical angina’. Anginal symptoms that arise spontaneously are typical of coronary spasm. Age, sex, ethnicity, vascular risk factors (e.g. cigarette smoking, obesity), the environment (e.g. air pollution), and mental health, influence symptoms in individualized ways. Other causes of ischaemic symptoms include coronary vasomotion disorders (i.e. microvascular disease and coronary spasm),^1^ myocardial disease (e.g. hypertrophy), and systemic disorders (e.g. anaemia, hypertension, and renal disease).

A second priority is establishing the aetiology and whether symptoms are related to CAD. In this regard, computed tomography coronary angiography (CTCA) has strengths and limitations. Imaging of atherosclerosis is reliably achieved using non-invasive CTCA and invasive coronary angiography and to some extent by coronary magnetic resonance imaging. However, symptoms are more specifically assessed using a functional test. A diagnosis of coronary atherosclerosis serves as the basis for primary prevention including lifestyle measures and pharmacotherapy to modify risk factors and prognosis. Obstructive CAD should be treated with medical therapy and in patients with ischaemic symptoms, functional testing or invasive management coupled with physiological assessments should inform a decision for ischaemia-guided myocardial revascularization.

Computed tomography coronary angiography lacks resolution for small vessel disease. In order to diagnose microvascular angina, myocardial ischaemia testing using non-invasive techniques, e.g. stress testing with positron emission tomography or cardiovascular magnetic resonance (CMR), or invasive tests of coronary vascular function (functional coronary angiography), are required.

Most patients presenting to chest pain clinics do not have obstructive CAD,^2,3^ and most of these patients are women.^3–6^ An initial focus on excluding obstructive CAD leaves many patients with unexplained chest symptoms and uncertain management.^4–6^ The natural history of ischaemic heart disease (IHD) differs between men and women. Obstructive CAD is more likely in men^2,3^ whereas ischaemia with no obstructive coronary arteries (INOCA) (including microvascular angina and vasospastic angina) is more likely in women.^4,5^ In the prospective, all-comers CorMicA registry, which included 391 patients undergoing clinically indicated coronary angiography, compared with patients with obstructive CAD, physical limitation due to angina and quality of life were worse in patients with small vessel disease.^5^ Furthermore, use of functional tests in addition to angiography led to a reappraisal of the diagnosis and changes in treatment in half of the study population. The stratified medicine intervention in CorMicA linked test findings with mechanistically targeted therapy and improvements in symptoms and quality of life occurred over a 1-year period.^5^ In contrast, in the SCOT-HEART trial,^7^ angina and quality of life improved less in the CTCA-guided group compared with standard care. These results highlight that CTCA-guided management without functional testing is suboptimal for achieving symptom relief, in part because only a minority of individuals presenting with chest pain have obstructive CAD.

Clinical presentations may be caused by a stable chronic coronary syndrome^8^ or an acute coronary syndrome. IHD, CAD, and coronary heart disease (CHD) are not synonymous terms and should not be used interchangeably.^8^ They should be considered in a hierarchical classification system. IHD is positioned at the highest level, and the causes categorized as disease-specific subgroups (i.e. endotypes). Second-order terms include CHD and INOCA, and third-order terms are the specific causes of angina i.e. native CAD, restenosis, or endotypes of INOCA, e.g. microvascular angina and vasospastic angina, and non-coronary endotypes, e.g. left ventricular hypertrophy. A patient may have multiple pathologies. Standardized nomenclature is an important premise for unbiased decision-making.^8^

Advances in medical technologies create new diagnostic possibilities. The data provided by an exercise test are clinically useful and prognostically validated^9^ but with limitations in test sensitivity and specificity for CAD. Advances in anatomical imaging of CAD using CTCA and in functional imaging of myocardial ischaemia using stress echocardiography, CMR, and nuclear imaging, are preferred options. Nonetheless, the treadmill exercise test remains clinically useful to assess a patient’s response to exercise, the reasons for ending a test, and the occurrence of symptoms and signs of ischaemia.^10^

The question arises for clinicians (and their patients) ‘Which test to choose’ and for healthcare funders ‘Which scanner (or service) to provide?’ In the past decade, multiple clinical trials have been published and practice guidelines have followed.^9,11,12^ Other factors are deterministic for end-user adoption ‘in the clinic’. They include the cost of the technologies, their availability, contrast agents and staff expertise. Exposure to ionizing radiation is uniquely caused by CTCA and nuclear imaging, and the lifetime risk of cancer is relevant for younger patients and women. About 1 in 10 patients are unsuitable for CTCA due to atrial fibrillation, body size, coronary calcification, e.g. in the elderly, and intolerance of beta-blockers, i.e. asthma and left ventricular dysfunction.

Should clinicians adopt a default anatomical strategy or a more individualized approach? The anatomical approach using CTCA is recommended in the UK National Institute for Health and Care Excellence (NICE) clinical guideline 95.^11^ Personalized medicine takes account of the patient’s characteristics to stratify management and this approach is endorsed in the more recent clinical guidelines from the European Society of Cardiology^9^ and North American guidelines.^12^

The editors of the *European Heart Journal* have posed the motion: ‘*Great Debate: CTCA should be the initial diagnostic test in suspected angina*’. The pros and cons of this strategy are summarized in *Table 1*. Professor Kramer and colleagues write for the motion and Professor Kunadian and colleagues write against it. We hope you agree that the authors have captured the key issues.

**Table 1:** The pros and cons of a computed tomography coronary angiography-first approach

Pros
Diagnosis of coronary atherosclerosis (high sensitivity) to inform the decision for preventive medical therapy and improve prognosis
FFR_CT_ provides data on the functional significance of coronary atherosclerosis increasing specificity for flow-limiting coronary artery disease, optimizing the decision for invasive management.
Incidental findings (cardiac and thoracic)
Scan generally well tolerated by patients
Brief scan duration facilitates ‘high throughput’ clinical service imaging
Cons
Excluded patients—arrhythmias, tachycardia, severe renal dysfunction, contraindications to beta-blocker—asthma, heart block
Heart rate control—prescription of beta-blocker or rate-limiting calcium channel blocker entailing physician and pharmacy visits before the hospital visit for the CTCA scan
Potential for contrast media reaction
Ionizing radiation exposure
In stable populations referred for CTCA, most individuals do not have obstructive coronary artery disease, leaving the diagnosis and onward management of symptoms uncertain in many referred patients
No data for microvascular function or myocardial ischaemia
Limited specificity for quantifying lumen loss due to atherosclerosis (moderate specificity leading to false positive results), especially within coronary calcification and stents
FFR_CT_ exclusion criteria include history of coronary revascularization, atrial fibrillation
In patients with persisting symptoms and no obstructive CAD, additional visits for downstream functional tests may be necessary, extending the care pathway
In ACS, a CTCA-first strategy has no prognostic benefit, prolongs hospital stay, increases hospital costs
Clinical service: the CTCA scan and report are usually not provided during the initial clinic visit, hence repeated visits are needed
FFR_CT_ adds to initial costs; downstream and overall costs may not increase
Cost-effectiveness uncertain, e.g. SCOT-HEART health economics analysis not available

ACS, acute coronary syndrome; CAD, coronary artery disease; CTCA, computed tomography coronary angiography; FFR_CT_, computed tomography-derived fractional flow reserve.

### Conflict of interest

C.B. is employed by the University of Glasgow which holds consultancy and/or research agreements with companies that have commercial interests in the diagnosis and treatment of ischaemic heart disease. The companies include Abbott Vascular, AstraZeneca, Boehringer Ingelheim, GSK, HeartFlow, Menarini Farmaceutica, Neovasc, Siemens Healthcare and Valo Health. C.B. acknowledges research support from the British Heart Foundation (PG/17/2532884; FS/17/26/32744; RE/18/6134217) and Medical Research Council (MR/S005714/1).

## Figures and Tables

**Table 1:** Key limitations of the SCOT-HEART and PROMISE trials

No.	Trial limitations	Impact
**SCOT-HEART**		
1.	Layered strategy combining anatomical CTCA data and functional ETT data instead of only early CTCA	Trial does not explore role of CTCA as first strategy but rather the role of the layered strategy
2.	Low use of functional testing	Not representative of standard of care in most centres
3.	No pre-specified guiding protocols for CTCA in both trial arms	Unclear explanation of how CTCA improved medical therapy over ETT alone
4.	Concern of patients treated sub-optimally in the standard care arm	Risk of bias against standard care arm
5.	Long-term outcome data only extracted from electronic records with no formal event adjudication	Risk of bias as potential outcome events were not evaluated by independent and blinded experts
6.	Pre-specified cost-effectiveness analysis not yet published	Cost-effectiveness of CTCA in the trial unknown
**PROMISE**		
1.	Ratio of functional testing strategies was focused more on reflecting current practice conditions in trial sites	Trial does not provide definitive insight into the harm or benefits of CTCA compared with functional testing
2.	More advanced functional methods such as PET or CMR were either under-represented or not used	Low or no representation of PET or CMR, and the lack of imaging in ETT (10%) may have caused bias against the functional testing arm
3.	Study does not explore test utility or performance	Utility and performance values of testing strategies unknown

CTCA, computed tomography coronary angiography; ETT, exercise tolerance test; PET, positron emission tomography; CMR, cardiac magnetic resonance; SPECT, single photon emission computed tomography.

**Table 2:** Technical factors affecting role of CTCA as a widespread first-line test

No.	CTCA technical limitations
1.	Non-diagnostic studies due to extensive coronary calcification in older and diabetic patients
2.	Risk of nephrotoxicity from iodinated contrast media
3.	Lower resolution for small vessel calibres and inability to assess microvasculature
4.	Artefacts from previous stents, sternal wires from CABG, and other metallic implanted devices
5.	Exposure to ionizing radiation
6.	Limited availability of CTCA capable scanners and certified practitioners globally

CTCA, computed tomography coronary angiography; CABG, coronary artery bypass grafting.

**Table 3:** Functional testing vs. CTCA

Functional	CTCA
**In diagnosis of symptom aetiology**	
• Higher test specificity for CAD	• Lower test specificity for CAD
• Excellent in assessing microvascular causes (INOCA, MINOCA)	• Limited ability to assess microvascular or vasospastic causes (INOCA, MINOCA)
• Capable of detecting pericardial and myocardial inflammatory diseases	• Less sensitive to detecting pericardial and myocardial inflammatory diseases
• Reliable in patients with extensive calcification, previous coronary stenting, CABG, and implanted devices	• Less robust in patients with extensive calcification, previous coronary stenting, CABG, and implanted devices
**In predicting prognosis**	
• Extensive prognostic evidence by the extents of ischaemic burden and viability to assess risk of adverse outcomes	• Extremely low patient risk of serious heart events if normal CTCA, but risk is variable in abnormal CTCA
**In guiding intervention**	
• Less associated with unnecessary ICA	• Associated with an overuse of ICA, which is important in the current era of decreasing CAD prevalence
• All functional modalities have reasonable clinical adaptation in guiding intervention	• FFR_CT_ is not well adapted clinically, costly, and with limited evidence in effective guidance of intervention
• Able to detect and size the extents of ischaemic burden and viability to guide invasive coronary referral	• Not able to detect size and extent of ischaemia and myocardial viability to guide further invasive coronary referral

CTCA, computed tomography coronary angiography; CAD, coronary artery disease; FFR_CT_, computed tomography-derived fractional flow reserve; INOCA, ischaemia with non-obstructive coronary arteries; CABG, coronary artery bypass grafting; ICA, invasive coronary angiography.

**Figure 1:**
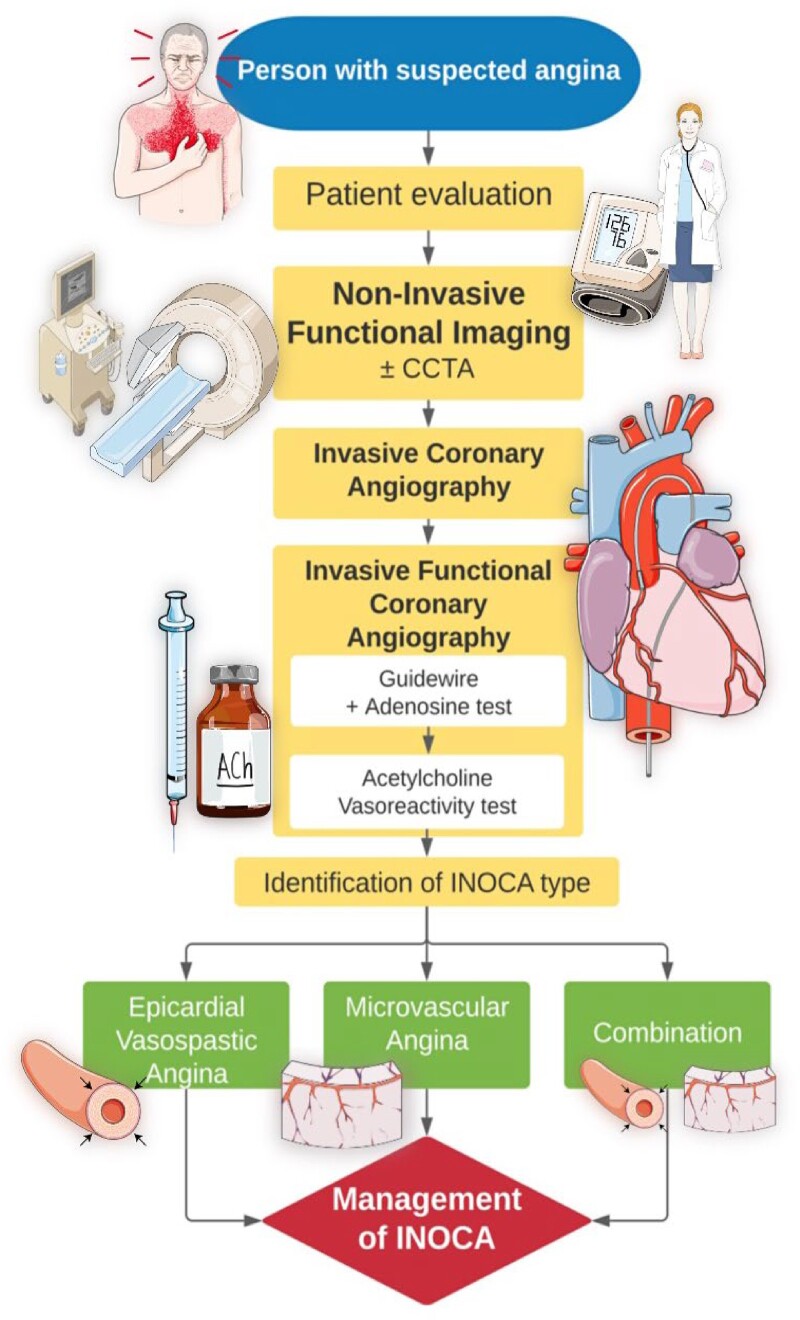
Diagnostic pathway for evaluation of ischaemia with non-obstructive coronary arteries (INOCA) according to the EAPCI expert consensus document. Investigation begins with non-invasive testing followed by invasive coronary angiography. CCTA: coronary computed tomography angiography.

## Pro

https://orcid.org/0000-0002-8558-7407PatelToral R
Cardiovascular Division, Department of Medicine, University of Virginia Health System, 1215 Lee St., Box 800158, Charlottesville, VA 22908, USAhttps://orcid.org/0000-0003-2674-3702VillinesTodd
Cardiovascular Division, Department of Medicine, University of Virginia Health System, 1215 Lee St., Box 800158, Charlottesville, VA 22908, USAhttps://orcid.org/0000-0003-2057-7731KramerChristopher M
Cardiovascular Division, Department of Medicine, University of Virginia Health System, 1215 Lee St., Box 800158, Charlottesville, VA 22908, USA
Department of Radiology and Medical Imaging, University of Virginia Health System, 1215 Lee St., Box 800170, Charlottesville, VA 22908, USA

Corresponding author. Tel: +1 (434) 982 0853, Fax: +1 (434) 982 1998, Email: ckramer@virginia

### Diagnostic value for assessment of stable angina

One of the most common assessments in daily practice is chest pain in patients with no known coronary artery disease (CAD).^1^ Over the past two decades, computed tomography coronary angiography (CTCA) has risen to the forefront for both the diagnosis and assessment of prognosis of CAD.^2^ Computed tomography coronary angiography is a fast efficient exam with a high sensitivity, high negative predictive value, and reasonable specificity for angiographically significant CAD.^3^ In an outpatient clinic setting, the SCOT-HEART trial demonstrated that the addition of CTCA was associated with improved diagnostic certainty of CAD and improved clinical outcomes compared with a non-computed tomography (CT) testing strategy in stable symptomatic patients.^4^ The PROMISE trial also demonstrated that CTCA is clinically useful as an alternative to functional testing in low–intermediate risk patients.^5^

The ISCHEMIA trial used CTCA to exclude left main artery (LM) disease (>50% stenosis severity) or non-obstructive CAD (stenosis <50%) in an effort to minimize the inclusion of patients without significant CAD.^6^ Importantly, one in five patients referred for enrolment in ISCHEAMIA following functional stress testing showing at least moderate ischaemia had no significant stenosis on CTCA. This highlights the significant limitations among the functional tests most utilized in this trial (stress ECG, stress nuclear myocardial perfusion imaging, and stress echocardiography) in selecting patients for invasive angiography. In a *post hoc* ISCHAEMIA analysis, pre-randomization CTCA studies demonstrated a high degree of concordance for CAD severity as compared with those who underwent subsequent invasive coronary angiography (ICA).^7^ Thus, CTCA is able to confidently exclude patients with LM (>50% stenosis) or high-risk CAD, as well as identify patients without any significant CAD, scenarios where ICA and revascularization are of unlikely benefit and where medical therapy is preferred.

This poses the question, should CT or functional imaging be the first-line test in patients with stable angina? With advancements in CT technology, plaque and stenosis characterization and identification of high-risk lesions [positive remodelling, low-attenuation (<30 HU), napkin ring sign], CTCA may improve the prognostic information and guide intensity of preventive therapies as compared with functional tests that only detect CAD at a later stage.^8,9,10^ In the CTCA group in the SCOT-HEART trial, a higher primary composite endpoint was noted when vulnerable plaque features were present [hazard ratio (HR) 3.01, 95% confidence interval CI 1.61–5.63].^8^ The findings in CONFIRM^11,12^ and PROMISE^5,13,14^ were similar as patients with high-risk plaque characteristics on CTCA had more frequent adverse events (HR 2.74, 95% CI 2.12–3.51). Quantitative CTCA, now clinically available, identifies total coronary plaque volume, per cent atheroma volume (the proportion of total vessel wall volume occupied by atherosclerotic plaque) and/or plaque volume indexed to coronary or patient size which have been reported to improve diagnostic and prognostic implications.^15,16^ Williams *et al*.^17^ showed that low attenuation plaque volume best predicted risk of subsequent myocardial infarction (MI) (HR 1.60, 95% CI 1.10–2.34) and patients with low attenuation plaque burden of >4% were five times more likely to suffer a fatal or non-fatal MI compared with those patients with less of a burden (HR 4.65, 95% CI 2.06–10.5).

CTCA offers significant advantages in the clinical setting compared with coronary artery calcium (CAC) scoring alone. These advantages include stenosis quantification, high-risk plaque identification and characterization, and improved risk stratification, especially in women who are known to exhibit more non-calcific plaque and lower calcium scores when compared with men of similar ages and risk factors.^18^ A meta-analysis of CTCA compared with functional imaging also concluded that the amount and type of atherosclerosis is the most prognostically useful imaging measure of individual patient risk and is more beneficial than identification of ischaemia during primary evaluation.^19^

In addition to ruling out high-risk coronary disease, CT-derived fractional flow reserve (FFR_CT_), a non-invasive physiological estimation of the effect of plaque burden, stenosis, and luminal diameter on coronary physiology, has been shown to improve the specificity of CTCA, with high accuracy compared with invasive FFR. The DeFACTO trial^20^ is one of numerous multicentre studies comparing FFR_CT_ to invasive FFR, with results showing improved diagnostic accuracy for detecting lesion-specific ischaemia, with sensitivity and specificity of FFR_CT_ of 74% and 67%, respectively. The area under the receiver operating characteristic curve (AUC) for FFR_CT_ was approximately 20% better at 0.81 vs. 0.50 for CTCA signifying the ability of FFR_CT_ to better discriminate lesion-specific ischaemia as compared with stenosis alone.^21,22^ In patients with multivessel CAD, FFR_CT_ is non-inferior to ICA and invasive FFR for decision-making.^19–23^

How does the use of CTCA affect management of patients and long-term outcomes? While the PROMISE trial did not show a significant benefit at 2 years of CTCA vs. conventional ischaemia testing, those randomized to CTCA had a lower rate of MI at 1 year and a significantly lower rate of normal cardiac catheterization in those referred for ICA.^5,13,14^ The use of preventative medical therapies increased in the SCOT-HEART trial in those who underwent CTCA and were sustained over the 5 years of follow-up. These treatments were selectively prescribed to patients who had CAD documented on CTCA despite comparable 10-year cardiovascular risk scores.^24,25^ By 5 years, there was a reduction in CHD death or non-fatal MI among those patients who underwent CTCA compared with standard care alone (48 [2.3%] vs. 81 [3.9%]; HR 0.59, 95% CI 0.41–0.84; *P* = 0.004) likely related to more intensive medical therapy for those with CAD identified using CTCA.^24^ While improving diagnosis, treatment, and CAD outcomes, CTCA was associated with a small attenuation of the improvements in symptoms and quality of life seen in the standard of care arm primarily due to patients with non-obstructive CAD requiring additional preventive medications.^26^

Considering the effect of CTCA on downstream use of ICA, several studies have shown that a CTCA first approach reduces the occurrence of a normal ICA.^10,13,15,23,25–28^ For example, in PROMISE, the proportion of patients with obstructive CAD on subsequent ICA was 72.1% compared with 47.5% in the functional group.^5^ In SCOT-HEART, there was no difference in rates of ICA, however, CTCA resulted in a higher rate of detection of obstructive coronary heart disease (79%), as confirmed by ICA than standard care alone (24%).^4^ Data from the ACC National Cardiovascular Data Registry have shown that using functional testing, approximately half of patients sent to ICA have no significant CAD.^29^ Thus, CTCA may provide improved patient selectivity for catheterization and, in the post-ISCHEMIA trial era, the opportunity to maximize medical therapies and lower costs.^30,31^

Functional tests are useful to identify microvascular ischaemia in the absence of obstructive coronary artery disease (INOCA). It is important to note that in order to make the diagnosis of INOCA due to microvascular disease, according to recently published ESC expert consensus statement, exclusion of obstructive epicardial CAD using CTCA or ICA is required.^32^ Hence, CTCA plays a central role in the diagnostic evaluation of patients with suspected microvascular disease, allowing for the use of PET or CMR (to document coronary flow reserve and myocardial blood flow) in selected, persistently symptomatic patients with non-obstructive CAD on CTCA.^32^

### Diagnostic value for assessment of angina in acute coronary syndromes

Chest pain evaluation is the second most common cause of emergency department admissions in the USA with nearly 6.5 million such evaluations each year. The evidence supporting the use of CTCA in patients presenting to the emergency department with acute chest pain is based on multiple large-scale, multicentre comparative effectiveness trials. Such randomized controlled trials have shown that a negative CTCA (normal or <50% stenosis) is associated with low cardiac event rates, lower rates of hospital admissions, and shorter lengths of stay when compared with standard of care, translating to cost-effective resource utilization and efficient care.^33,34^ Importantly, the detection of CAD (obstructive, but especially non-obstructive) was also significantly higher in patients who underwent CTCA in these trials, allowing for greater initiation of cardiovascular preventive treatments.^22,34^ The ROMICAT-II trial was also notable for a higher proportion of patients assigned to CTCA who were directly discharged from the emergency department (47% vs. 12%; *P* < 0.001).^34^ These trials made a strong argument for CTCA to be used in intermediate risk patients, which is now reflected in international guidelines. Specifically, the 2020 ESC guidelines for acute coronary syndromes notes a Class IA recommendation for the use of CTCA in low-intermediate risk patients with equivocal high-sensitivity troponin values or non-diagnostic prior functional tests and in patients with negative biomarkers and ECG but ongoing clinical concern for acute coronary syndrome.^35^ Similarly, as stated above, in the US multisocietal chest pain guideline, CTCA is now a first-line test for patients with acute chest pain at intermediate risk using high-sensitivity troponin and clinical decision pathways who do not have known CAD.^36^

It is important to note that the use of CTCA in acute chest pain has been predominately validated in low-to-intermediate risk patients according to initial troponin and/or clinical decision pathways. Current guidelines cited above recommend the use of CTCA in patients without known CAD, negative or equivocal troponin, and low risk ECG findings. The recently published RAPID-CTCA trial compared CTCA (majority done within 72 h of admission) vs. standard of care in the UK among 1748 patients with acute chest pain and at least 1 or more of the following: abnormal troponin elevation, prior CAD, or abnormal ECG.^37^ Importantly, 34% had known CAD and 49% were assessed ultimately to have ACS. Computed tomography coronary angiography did not improve clinical outcomes.

### International guidelines

Recent guidelines now recommend CTCA as a first-line testing option in patients with acute or chronic chest syndromes who do not have known CAD. The 2019 ESC chronic coronary syndrome guideline recommended the use of either non-invasive functional imaging for ischaemia (Class I) or anatomical imaging using CTCA (Class I) as an initial test for diagnosing CAD.^3^ The ESC guidelines also gives a Class IIa recommendation to CTCA as a ‘gatekeeper’ to the catheterization lab in patients with equivocal or mildly abnormal ischaemia tests. The most recent ACC/AHA multisocietal chest pain guideline emphasized the need for a focused history in classifying chest pain as cardiac, possibly cardiac, or non-cardiac and risk stratification with pre-test probability to determine in whom further diagnostic testing is appropriate.^37^ For the majority of patients at low risk (<15% pre-test probability) with possibly cardiac or non-cardiac chest pain, CAD testing is optional and shared decision making with patients is encouraged. The guideline gave CTCA a Class I, level of evidence A recommendation for intermediate risk patients with acute chest pain and no CAD and chronic stable chest pain with no known CAD. Additionally, CTCA is also recommended as a ‘gatekeeper’ study to ICA, recommended (IIa) in non-high-risk patients with mildly abnormal or equivocal abnormalities on functional ischaemic testing, and reasonable (IIa) among patients with known non-obstructive CAD and stable chest pain despite guideline-directed medical therapy.^36^

### Conclusion

Non-invasive cardiac testing in patients with chronic or acute chest pain should shift to CTCA as a first-line strategy in appropriate patients given its strong negative predictive value for obstructive CAD, proven ability to identify prognostically important non-obstructive CAD, and relatively low cost. Current international guidelines designate CTCA a first-line test for patients presenting with acute or chronic chest pain who are without known CAD based on comparative effectiveness studies showing that CTCA improves the detection of clinically relevant CAD and increases utilization of appropriate medical therapy.

## Contra

https://orcid.org/0000-0001-8212-0759KwongRaymond Y
Cardiovascular Division, Department of Medicine, Brigham and Women’s Hospital and Harvard Medical School, Boston, MA, USARaharjoDaniell Edward
Translational and Clinical Research Institute, Faculty of Medical Sciences, Newcastle University, 4th Floor William Leech Building, Newcastle upon Tyne NE2 4HH, UK
Cardiothoracic Centre, Freeman Hospital, Newcastle upon Tyne Hospitals NHS Foundation Trust, Newcastle upon Tyne, UKhttps://orcid.org/0000-0003-2975-6971KunadianVijay
Translational and Clinical Research Institute, Faculty of Medical Sciences, Newcastle University, 4th Floor William Leech Building, Newcastle upon Tyne NE2 4HH, UK
Cardiothoracic Centre, Freeman Hospital, Newcastle upon Tyne Hospitals NHS Foundation Trust, Newcastle upon Tyne, UK

Corresponding author. Tel: 0044 191 2085797, Email: vijay.kunadian@newcastle.ac.uk

In this debate, we present the opposing views as to why there are challenges using computed tomography coronary angiography (CTCA) as a first-line test for chest pain evaluation and discuss the need for selective use of CTCA to inform decisions and guide management in patients with suspected coronary artery disease (CAD) in the stable and acute settings (*Graphical Abstract*).

### Diagnostic value for assessment of stable angina

The initial evaluation of stable chest pain patients should be practical, safe, cost-effective, accurate in diagnosing aetiology, and effective in guiding therapy. In the PROMISE trial, the CTCA-first strategy did not reduce the composite primary endpoint, its components [death, myocardial infarction (MI), unstable angina hospitalization, and procedural complication], or healthcare costs compared with functional testing at a 2-year follow-up.^1^ The CTCA group had a higher 90-day referral rate to invasive coronary angiography (ICA) (12.2% vs. 8.1%), coronary revascularization (6.2% vs. 3.2%), and overall exposure to medical radiation compared with functional testing (12.0 ± 8.5 vs. 10.1 ± 9.0 mSv, *P* < 0.001).^1^ In contrast, the SCOT-HEART trial of 4146 chest pain patients reported a reduction of non-fatal MI (*n* = 44 [2.1%] vs. *n* = 73 [3.5%]) after 5 years despite initially demonstrating no difference in outcomes.^2^ The SCOT-HEART and PROMISE trials had strengths and limitations (*Table 1*).

Both the PROMISE and SCOT-HEART trials studied low or low-intermediate risk patients and indeed demonstrated that CTCA is useful in ruling out CAD in patients from these pre-test risk groups; however, anatomical CTCA does not perform as robustly in ruling out CAD in intermediate-high risk symptomatic patients.^3^ Given the prevalence of obstructive CAD in symptomatic chest pain patients is generally low (<10%), anatomical imaging by a CTCA-first strategy has a lower test specificity for significant stenosis that warrants coronary intervention, in comparison with most non-invasive functioning test.^4^ In the Ce-MARC 2 trial (*n* = 1202), CTCA imaging was included as per the 2010 NICE guideline arm if the pre-test likelihood of coronary heart disease was <30%. The investigators reported that NICE-guideline-directed care at the time of the trial was associated with the occurrence of unnecessary ICA at 12 months in 29% of the participants, compared with 8% of participants in the functional imaging-first strategy (by cardiac magnetic resonance [CMR]/myocardial perfusion scintigraphy).^5^ Another prospective analysis showed that CTCA led to subsequent ICA overuse with almost 50% not resulting in revascularization.^6^ Some CTCA proponents have suggested that computed tomography-based fractional flow reserve (FFR_CT_) integrates anatomy and luminal physiology as a ‘one-stop-shop’ to guide ICA referral.^7^ However, FFR_CT_ quantitation involves assumptions in its fluid dynamics computational models, requires artefact-free imaging, and is not robust in patients with atrial fibrillation, prior coronary stenting or extensive coronary calcification, severe valvular heart disease, sequential luminal lesions, or prior coronary bypass. As a result, between 20% and 40% of CTCA studies may be deemed inadequate for FFR_CT_ analysis. Second, the correlation of FFR_CT_ with invasive FFR is not high in contrast with the established evidence by functional imaging such as CMR or positron emission tomography (PET). In a meta-analysis using invasive FFR as the reference standard, a FFR_CT_ cut-off of 0.73 only achieved a 50% per-vessel agreement with invasive FFR. When a diagnostic threshold of 95% was set, only FFR_CT_ values measured to be in the extreme ends (below 0.53 or above 0.93) met this threshold.^8^ Third, clinical adaptation and prognostic implications of FFR_CT_ are both substantially more limited than any one of the conventional functional imaging methods. Logistically, the only approved method at present is an off-site solution offered by a commercial vendor (HeartFlow, Redwood City, CA, USA) at approximately £530 (∼620 Euros) per analysis in addition to the £220 (∼258 Euros) per NHS cost of the CTCA scan.^9–11^ In the FORECAST trial (*n* = 1400), compared with standard care (in which CTCA predominantly was the first test), FFR_CT_-guided care did not result in benefits in healthcare costs, cardiovascular outcomes, or quality of life.^12^ FFR_CT_ did reduce the use of ICA but the comparative group was primarily CTCA itself, not functional imaging. Therefore, additional high-quality clinical evidence prior to more widespread implementation is warranted and there is no convincing evidence to promote a CTCA-first strategy for all patients. Several factors may reduce the diagnostic performance and widespread adaptability of a CTCA-first strategy (*Table 2*).

All stress modalities have shown that an abnormal test is associated with adverse outcomes, but evidence of ischaemia does not imply causality. Advanced functional imaging, most notably stress CMR and PET, has extensive established evidence of providing diagnostic and prognosticating values across a broad spectrum of stable chest pain patients. Both stress CMR and PET accurately detect size and extent of ischaemic burden and viability which are robust non-invasive risk markers that guide invasive coronary referral. In randomized clinical trials to date, revascularization based on ischaemia has not improved clinical outcomes.^13^ However, physiologic evidence of ischaemia correlates reasonably well with patients’ symptoms and may guide the use of invasive coronary intervention towards improvement of symptoms.^14,15^ Both stress CMR and PET have demonstrated high sensitivity and specificity when correlated to invasive FFR and thus provide the capacity for non-invasive guidance of coronary revascularization based on physiologic significance of CAD.^16,17^ The MR-INFORM trial randomized 918 intermediate-high risk stable chest pain patients to stress CMR-first vs. invasive FFR-first strategies, and observed effective reduction of 20% of coronary revascularization (36% vs. 45%, *P* = 0.005) by the CMR-first approach without compromising patient safety or worsening symptoms at one year.^18^ Many large registries, including the recent Stress CMR Perfusion Imaging in the United States (SPINS) registry,^19^ have reported effective risk differentiation by stress CMR: patients with absence of ischaemia or clinically unrecognized infarction consistently experienced very low cardiac event rates and its use was cost-effective. In the STRATEGY trial, 600 patients with stable chest pain were randomized to stress CMR vs. CTCA.^20^ After a median follow-up of 2 years, stress CMR guidance was associated with lower adverse cardiac events (5% vs. 10%, *P* < 0.01), in addition to lower downstream testing costs, coronary revascularization, and medical radiation exposure (*Table 3*).

With validated quantitation of myocardial blood flows and reserve, functional imaging by PET and CMR have unique roles in assessing chest pain patients with ischaemia with no obstructive coronary arteries (INOCA).^21^ Up to 50% of patients undergoing ICA due to chronic coronary syndrome and evidence of ischaemia, especially prevalent among women and diabetics, reveal INOCA.^22^ An anatomical CTCA-first strategy will lead to systematic under-diagnosis of coronary microvascular and vasospastic angina, particularly relevant to women.^23^ Consequently, sub-optimal therapy may be provided to patients resulting in persistent angina, impaired quality of life, and worse short- or long-term cardiovascular outcomes.^24^ In a CTCA first-line approach, most of these patients will need further cardiac testing, increasing downstream resource utilization^25–27^ and potentially repeated exposure to iatrogenic radiation. This hinders efficient clinical management, increases iatrogenic morbidity, and raises societal healthcare costs. Functional imaging by stress CMR and PET have demonstrated important contributions in assessing INOCA, although these techniques are not useful for eliciting vasospasm.^21^ In addition to excluding obstructive CAD using CTCA or ICA, the presence of reversible ischaemia should be established according to an expert consensus of INOCA.^24^ A well-tailored management strategy can then be provided to patients once a diagnosis has been established (*Figure 1*). Furthermore, CMR has other advantages over CTCA as it routinely diagnoses pericardial and myocardial inflammatory diseases as alternative aetiologies for chest pain symptoms.^28^

### Diagnostic value for assessment of angina in acute coronary syndromes

The recently published RAPID-CTCA trial further discredits the utility of CTCA as an all-purpose solution for first-line angina assessment. This trial revealed no significant difference in the primary outcomes between the CTCA and non-CTCA groups (CTCA: 5.8%, non-CTCA: 6.1%, *P* = 0.65) among low-intermediate-risk patients with suspected or provisional acute coronary syndrome. The median hospitalization length of stay was 2.2 days in the CTCA group vs. 2.0 days in the usual care group. Median costs were also higher by US$718 per patient assessed early on with CTCA.^29^ The results demonstrate how early CTCA does not improve clinical outcomes or length of hospitalization, increases healthcare costs and may cause over-testing. This further corroborates the notion that the first-line assessment should be personalized.

In the context of acute coronary syndrome, MI with non-obstructive coronaries (MINOCA) occurs in ∼8–15% of patients with a clinical diagnosis of MI. CMR plays an important role in the identification of the causes (plaque rupture/erosion, Takotsubo cardiomyopathy, myocarditis) of MINOCA.^30^ CMR can assess both cardiac structures, function and tissue characteristics. Patients with a history of acute chest pain followed by a normal stress CMR result have an excellent short- and mid-term prognosis.^31^ In patients presenting with an non-ST-elevation MI of uncertain aetiology, a CMR strategy may identify the underlying cause and reduce the need for ICA.^32^ Other non-invasive diagnostic tests such as stress echocardiography, exercise ECG, stress PET/single photon emission computed tomography (SPECT) myocardial perfusion imaging may be utilised for medically stabilized patients with acute chest pain.

### International guidelines

The 2021 AHA/ACC chest pain guidelines recommended numerous Class 1 and Class 2A indications for use, generally and equally represented for functional imaging (stress CMR, SPECT/PET, stress echocardiography) and anatomical CTCA, across stable and acute chest pain syndromes.^33^ The guideline specifically emphasized that physicians should base their choices of non-invasive tests on individualized clinical risk assessment and patient characteristics (e.g. pre-test risk of CAD based on age, sex, and symptomatology, risk of the procedure, ability to exercise and reliability of baseline ECG, and radiation burden), local expertise/availability, and patient preferences. There is no recommendation of a CTCA-first approach broadly in either stable or acute chest pain syndromes; however, some specific mentioning of preferred tests of choice were made. For patients with stable chest pain with no known CAD at intermediate/high risk of obstructive CAD, both CTCA or functional imaging-based tests (stress CMR, SPECT/PET or echocardiography) received a Class 1 recommendations for use. In patients at low pre-test risk, no testing is recommended as Class I while it is reasonable (Class 2a) to consider either a coronary calcium score or an exercise ECG test, and neither CTCA nor any of the functional imaging tests was recommended.^33^ For patients with stable chest pain with known CAD, the guideline emphasized the need to evaluate clinical responses to intensified medical therapies and frequency/urgency of symptoms, and recommended the use of either functional testing (Class 1 or Class 2a) or CTCA (Class 2a) to be both appropriate. For patients with stable chest pain, the guideline also highlighted the increasing need of awareness of INOCA. In patients suspected to have INOCA, stress CMR and PET were recommended (Class 2a) as validated non-invasive methods available to quantify absolute myocardial blood flow, along with a more comprehensive assessment by invasive functional testing (Class 2a).

Similarly, for patients with acute chest pain and no known CAD, both functional stress testing (including stress ECG) and CTCA have Class 1 recommendations and the choice of test should be dictated by clinical assessment. It was specifically mentioned that functional stress testing may be needed in those patients with inconclusive anatomical CTCA and vice versa (both Class 2a recommendations). For patients with acute chest pain and known CAD where defer testing is not suitable, risk assessment is of paramount importance thus functional stress imaging (excluding stress ECG) should be used regardless of prior knowledge of coronary obstruction (Class 2a). This contrasts with CTCA in this setting when it should be used only if CAD was known to be non-obstructive (Class 2a). In addition, by characterizing both cardiac structures and tissue characteristics, a non-stress CMR specifically has two additional Class 1 recommendations for patients presenting with acute chest pain. One for diagnosing the cause of acute chest pain in patients with myocardial injury but have no obstructive coronary disease (MINOCA). The other for determining the presence and extent of myocardial and pericardial inflammation and fibrosis in patients with suspected acute pericarditis. This is relevant in the current global pandemic when both the index viral infection and vaccinations may cause perimyocarditis.^34,35^

### Conclusion

CTCA is useful in assessing some patients with chest pain but limitations summarized in this review render its generalized first-line use for all patients inadvisable. Computed tomography coronary angiography should remain targeted for patients assessed to have lower likelihood of CAD due to significant limitations in patients with higher pre-test probability or established coronary heart disease. Clinical judgement to choose between CTCA, non-invasive functional testing, and ICA should be exercised in a personalized way.

### Data availability

No new data were generated or analysed in support of this manuscript.
